# Effect of Dipeptidyl Peptidase-4 (DPP-4) Inhibition on Biomarkers of Kidney Injury and Vascular Calcification in Diabetic Kidney Disease: A Randomized Controlled Trial

**DOI:** 10.1155/2021/7382620

**Published:** 2021-10-16

**Authors:** Thananda Trakarnvanich, Bancha Satirapoj, Swangjit Suraamornkul, Thanit Chirananthavat, Anoma Sanpatchayapong, Torpong Claimon

**Affiliations:** ^1^Faculty of Medicine, Vajira Hospital, Navamindradhiraj University, Bangkok, Thailand; ^2^Division of Nephrology, Department of Medicine, Phramongkutklao Hospital and College of Medicine, Bangkok, Thailand; ^3^Department of Medicine, Police General Hospital, Bangkok, Thailand

## Abstract

**Introduction:**

Dipeptidyl peptidase-4 (DPP-4) inhibitors improve glycemic control and have pleiotropic effects on kidney injury, albuminuria, and vascular inflammation, especially in animal models. We evaluated the effects of a potent DPP4 inhibitor (gemigliptin) on these processes among patients with diabetic kidney disease (DKD).

**Methods:**

This study employed a multicenter, prospective, randomized, placebo-controlled design. A total of 201 participants were enrolled and randomly assigned to one of two groups, one received treatment with 50 mg gemigliptin daily along with standard care for diabetes mellitus for 6 months. The changes in the coronary calcium score (CAC score), cardio-ankle vascular index (CAVI), estimated glomerular filtration rate (eGFR), vascular calcification level, and tubular renal injury marker expression were evaluated at baseline and 6 months.

**Results:**

In total, 182 patients completed the study. Significant reductions in hemoglobin A1C levels were observed in both groups. The changes in the CAC score, CAVI, eGFR, and level of proteinuria over the 6 months of the study did not significantly differ between the gemigliptin and control groups. However, biomarkers of vascular calcification, including serum bone alkaline phosphatase and kidney injury, including urine neutrophil gelatinase-associated lipocalin (NGAL)/Cr and urine liver fatty acid-binding protein (L-FABP)/Cr, were improved significantly in the gemigliptin treatment group compared with the control group. No serious adverse events were observed during the study.

**Conclusion:**

Our study showed that gemigliptin significantly improved the expression of renal tubular injury biomarkers and vascular calcification levels among patients with DKD; however, gemigliptin did not affect renal function or coronary calcification compared with those observed in the control. A larger study with a longer follow-up is essential to verify these beneficial effects. *Clinical Trials*. This trial is registered with ClinicalTrials.Gov Identifier NCT04705506.

## 1. Background

The advantage of dipeptidyl peptidase-4 (DPP-4) inhibitors is their lower risk of inducing hypoglycemia among patients with type 2 diabetes [1]. DPP-4 inhibitors impede the proteolytic enzyme DPP-4, resulting in delayed degradation of glucagon-like peptide I (GLP-1), thus improving glycemic control. Additionally, GLP-1 regulates the calcification of vascular smooth muscle cells through numerous pathways [2]. Gemigliptin is a potent DPP-4 inhibitor that has been approved for use among patients with type 2 diabetes and provides pleiotropic effects in addition to its glucose-lowering effects. It inhibits lipopolysaccharide- (LPS-) induced proinflammatory effects in vascular endothelial cells by attenuating NF-kappa B and c-Jun NH (2)-terminal kinase (JNK) signaling via an AMP-activated protein kinase- (AMPK-) dependent mechanism [3]. A recent study revealed that gemigliptin attenuates calcification of the abdominal aorta, RUNX2- and phosphate-induced Pit-1 mRNA expression, and reactive oxygen species formation [4]. Therefore, gemigliptin could alleviate vascular calcification among patients with a high risk of disease, especially diabetic kidney disease (DKD).

DPP-4 inhibitors can be used safely for patients with type 2 diabetes with renal impairment. Moreover, in an experimental model, DPP-4 inhibitors, especially gemigliptin, substantially decreased albuminuria and renal fibrosis in mice with unilateral ureteral obstruction [5] and attenuated podocyte injury in mice with diabetic nephropathy [6]. Recently, treatment with DPP-4 inhibitors attenuated kidney injury and improved acute and chronic kidney injury [7]. We therefore investigated whether gemigliptin has a similar effect among patients with DKD with a high level of vascular calcification and renal progression. We investigated the effect of gemigliptin on vascular calcification and renal injury among patients with type 2 diabetes with renal involvement.

## 2. Methods

### 2.1. Study Population

We prospectively enrolled patients from the following three hospitals in Bangkok, Thailand: Vajira Hospital, Police Hospital, and Phramongkutklao Hospital. The study implementation and protocol were approved by the institutional review board and adhered to the tenets of the Declaration of Helsinki. Informed consent was obtained from all the participants before enrollment. The inclusion criteria included patients with type 2 diabetes with CKD stages 3 to 4 (estimated glomerular filtration rate (eGFR) 15 to 60 mL/min/1.73 m^2^), persistent micro- or macroalbuminuria, and stable glycemic control for 12 weeks. The exclusion criteria included a history of allergy to DPP4 inhibitors, documented severe osteoporosis, concurrent infectious disease, inflammatory diseases, postkidney transplantation, and treatment with calcimimetic agents, bisphosphonates, GLP-1 receptor agonists, DPP4 inhibitors, and sodium-glucose cotransporter-2 inhibitors. Patients were withdrawn from the study when they experienced other serious side effects after inclusion, had severe hypoglycemia, or required hospitalization.

### 2.2. Study Design

**Figure 1:**
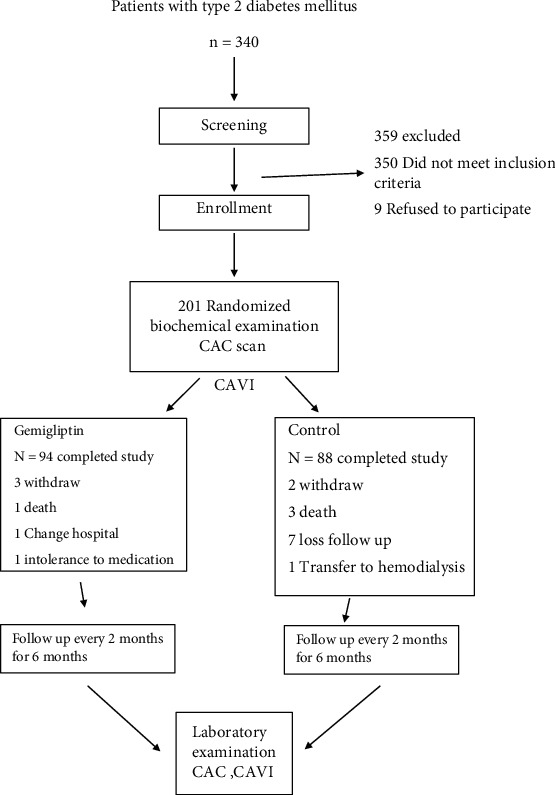
Enrollment, follow-up, and vital status.

This constituted a multicenter prospective, open-label, randomized controlled trial. Patients were randomized by the study coordinator in blocks of four; the allocation was concealed, and the patients were then divided in two groups at a ratio of 1 : 1. Group 1 received 50 mg gemigliptin daily for 6 months in addition to standard treatment, and group 2 received standard treatment for type 2 diabetes and CKD. Patients were scheduled for follow-up visits at months 2, 4, and 6, as shown in Figure 1. Diet and lifestyle modifications were advised for all participants. For 80% power at *P* < 0.05 to detect a difference in biomarker levels of vascular calcification after gemigliptin treatment among patients with CKD, a total of 100 patients per group were required. Overall, 182 participants were recruited [4].

From September 2018 to October 2020, we collected baseline data from all participants including demographic characteristics, comorbid conditions, and physical examinations. Blood samples were collected and allowed to clot for 30 min at room temperature before centrifugation for 15 min. The serum was stored at -20°C until assayed. Urine was collected in a sterile container, centrifuged to remove particulate matter, and stored at -20°C until assayed. Biochemistry indexes, including complete blood count, blood urea nitrogen (BUN), creatinine, fasting plasma glucose, hemoglobin A1c, calcium, phosphorus, albumin, and 24-hour urine protein, were measured at baseline and at each study visit.

The following were measured at baseline and after six months: the coronary artery calcium (CAC) score; cardio-ankle vascular index (CAVI); vascular calcification biomarker levels including serum osteopontin, bone alkaline phosphatase, and reactive oxygen species (myeloperoxidase); and renal injury biomarker levels including urine kidney injury molecule-1 (KIM-1), neutrophil gelatinase-associated lipocalin (NGAL), and liver fatty acid-binding protein (L-FABP). All vascular and renal injury biomarker levels were measured using the quantitative sandwich enzyme immunoassay technique (R&D Systems, USA) according to manufacturer instructions. Absorbance was measured at 450 to 570 nm using a microplate reader (Sunrise™ Absorbance Reader, TECAN, Switzerland). Urine creatinine was assayed using the enzymatic method (Architect C16000 analyzer, Temecula, CA, USA).

### 2.3. Coronary Artery Calcium Scoring (CAC Score)

All patients underwent computer tomography (CT) examination using an Ingenuity CT scanner (128 slices, Philips Medical Systems, Nederland B.V.), and images were acquired using axial prospective gating while breath holding. The calcium score of each lesion was calculated using the Agatston method [8]. The software package (Heart Beat Calcium Scoring, IntelliSpace Portal, version 7.0; Philips Medical Systems, Nederland B.V.) automatically calculated and displayed the CT attenuation values. Agatston scores were reported for each of the four major coronary arteries, and the sum of the scores for these arteries was reported.

### 2.4. Cardio-Ankle Vascular Index (CAVI)

We measured CAVI using a vascular screening system (Vasera VS-1500; Fukuda Denshi, Co. Ltd., Tokyo, Japan) with the patient lying supine and the head placed in the midline position. Cuffs were then applied bilaterally to the arms and ankles, and the patient was allowed to rest for 15 min. Measurements began by obtaining the blood pressure of the right brachial artery and ankle, followed by the left brachial artery and ankle. Pulse wave velocity was measured by dividing the vascular length by the time taken for the pulse wave to propagate from the aortic valve to the ankle. The ankle brachial pressure index was also calculated. A CAVI value < 8 was considered normal.

### 2.5. Statistical Analysis

Continuous and categorical variables are described as mean ± standard deviation and numbers with parentheses. Differences between groups were compared using Student's *t*-test and the chi-square test. The number and percentage of the variables are presented by treatment group, and differences between the two treatment groups were compared using Fisher's exact test. The comparison between variables before and after treatment with gemigliptin was performed using ANOVA. A multiple linear regression analysis was performed to evaluate the effects of HbA1c on renal injury markers. The level of significance for all statistical tests was set at 0.05. All analyses were performed using SPSS (IBM Corp., Armonk, NY, USA) for Windows Software, version 22.

## 3. Results

### 3.1. Patient Characteristics

Of the 260 patients screened for eligibility, 201 (77%) were enrolled in this randomized clinical trial. Of these participants, 6 were withdrawn due to personal reasons, and 13 were excluded for other reasons. The remaining 182 participants completed the study, as shown in Figure 1.

There were 107 males and 75 females included in this study. The mean age was 62.77 ± 9.59 years. Sixty-two patients (80.52%) and 28 patients (36.36%) presented hypertension and dyslipidemia, respectively. A total of 14.29% (*n* = 11) of the patients had a history of ischemic heart diseases, while 1 patient (1.3%) had a history of peripheral arterial disease. All patients were on oral antidiabetic agents with or without insulin. Over 95% of patients received background antidiabetic treatment with insulin (37.8%), sulfonylurea (71.1%), or other groups of oral hypoglycemic agents with insulin (29.9%) in the gemigliptin group. The equivalent proportion of patients in the control group received the same regimen of diabetes treatment. Treatment groups were balanced with respect to hypertensive medications used except for ACEi/ARB, which was more commonly used by patients in the gemigliptin group (63.3 vs. 46.0%, *P* = 0.02 in the gemigliptin and control groups, respectively). At baseline, the 182 patients had a mean HbA1c of 8.25 ± 1.83%, a mean estimated GFR of 45.58 ± 20.18 mL/min/1.73 m^2^, and a mean body mass index of 28.16 ± 5.37 kg/m^2^. The demographic and baseline characteristics of the groups were comparable, as shown in Table 1.

### 3.2. Glycemic Control

**Figure 2:**
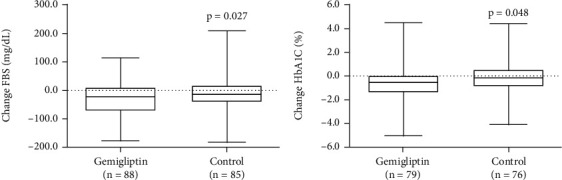
Box plot indicating changes in fasting blood sugar (a) and glycated hemoglobin (b) during the trial period. Data were set on the basis of scheduled visits.

Changes in the HbA1c values over time are shown in Tables 2 and 3. The mean HbA1c level decreased from 8.3 ± 1.95% at baseline to 7.7 ± 1.98% at six months (*P* < 0.001) in the gemigliptin group and from 8.12 ± 1.70% at baseline to 7.98 ± 1.88% at six months in the control group (*P* = 0.451) (Tables 3 and 4). Significant reductions in HbA1c levels were observed at month 6 in the gemigliptin group (-0.67%) compared with the HbA1c levels of the control group (-0.15%; *P* = 0.048) (Figure 2 and Table 4).

### 3.3. Gemigliptin and Vascular Calcification

The calcium content in the coronary wall, as measured by CAC scores, increased over time in both groups but did not significantly differ before and after the treatment period (the CAC score in the gemigliptin group increased from 655.72 ± 905.88 to 690.93 ± 932.89 (*P* = 0.18) and increased from 729.95 ± 1123.98 to 744.67 ± 1148.90 in the control group). The change in CAC scores was also nonsignificant between the two groups (Table 4).

### 3.4. Gemigliptin and Vascular Stiffness

We measured CAVI to assess arterial stiffness. After treatment, CAVI tended to improve in the gemigliptin group (9.37 ± 1.35 at baseline vs. 9.08 ± 1.52 at six months, *P* = 0.08), and CAVI significantly improved in the control group (9.26 ± 1.44 at baseline vs. 8.69 ± 2.06 at six months, *P* = 0.005). However, the change in CAVI did not differ between the two groups (*P* = 0.265) (Table 4).

### 3.5. Gemigliptin and Markers of Vascular Calcification

**Figure 3:**
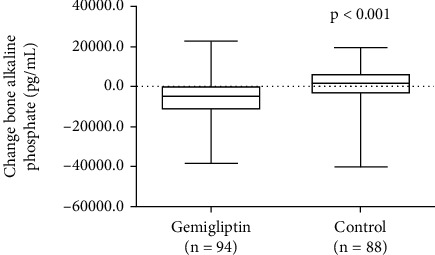
Changes in bone alkaline phosphatase in both groups at baseline and at the end of the study.

To examine whether gemigliptin provided protective effects against vascular calcification, we examined the biochemical markers involved in vascular calcification and oxidative stress. Serum osteopontin levels showed no significant differences from the baseline value in either group, and the mean changes did not significantly differ between the two groups (Table 4). The major bone mineralization regulator (bone alkaline phosphatase) decreased in the gemigliptin group but increased in the control group. At six months, the level of bone alkaline phosphatase significantly reduced in the gemigliptin treatment group compared with the control group (−5.84 ± 10.65 *μ*g/L vs. 0.08 ± 11.45 *μ*g/L, *P* < 0.001, respectively) (Table 4 and Figure 3). However, serum myeloperoxidase levels, indicating oxidative stress, did not change significantly from baseline, and the mean changes did not differ between the two groups.

### 3.6. Gemigliptin, Renal Function, and Proteinuria

To verify the short-term effect of gemigliptin on estimated GFR and proteinuria, the changes in estimated GFR from one point in the treatment timeline to the six-month time point were compared between the treatment and standard control groups. At baseline, 24-hour urine protein excretion in the gemigliptin group was higher than that in the control group (1.77 ± 3.15 vs. 0.89 ± 1.67 g/day, *P* = 0.048, respectively). The mean changes in estimated GFR and urine protein levels did not significantly differ from baseline in the gemigliptin and control groups.

### 3.7. Gemigliptin and Renal Injury Biomarkers

**Figure 4:**
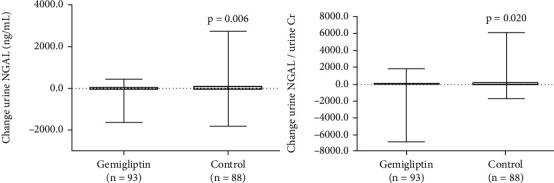
Comparisons between urine NGAL and urine NGAL factors for urine creatinine in both groups at baseline and at the end of the study.

We examined the effects of gemigliptin on the levels of urinary renal injury biomarkers, such as NGAL, L-FABP, and KIM-1. We adjusted urine biomarker concentrations using urine creatinine levels to eliminate the effects of patient hydration status. Urine NGAL levels tended to decrease but did not reach statistical significance in the gemigliptin group (387.9 ± 1094.02 ng/mg creatinine at baseline vs. 316.37 ± 679.96 ng/mg creatinine at the end of the study, *P* = 0.412) (Table 2). However, urine NGAL significantly increased in the control group (333.67 ± 627.23 ng/mg creatinine at baseline vs. 590.95 ± 1252.05 ng/mg creatinine at the end of the study, *P* = 0.024) (Table 3). The change in urine NGAL between the two groups significantly differed (−71.53 ± 837.30 ng/mg creatinine in the gemigliptin group vs. 257.28 ± 1047.29 ng/mg creatinine in the control group, *P* = 0.020) (Table 4 and Figure 4).

**Figure 5:**
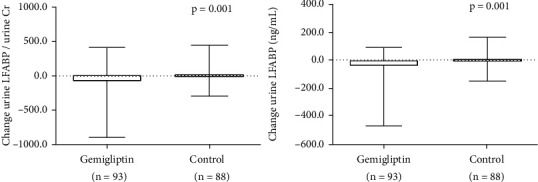
Comparisons between urine LFABP and urine LFABP factors for urine creatinine in both groups at baseline and at the end of the study.

Urine LFABP decreased significantly in the gemigliptin group (91.32 ± 146.92 *μ*g/mg creatinine at baseline vs. 37.16 ± 68.47 *μ*g/mg creatinine at the end of the study, *P* < 0.001), while urine LFABP was not significantly changed in the control group (48.86 ± 67.14 *μ*g/mg creatinine at baseline vs. 55.47 ± 86.31 *μ*g/mg creatinine at the end of the study, *P* = 0.488). The degree of change in the control group significantly differed from that in the gemigliptin group (−54.17 ± 141.64 *μ*g/mg creatinine in the gemigliptin group vs. 6.6 ± 88.94 *μ*g/mg creatinine in the control group, *P* = 0.001) (Table 4 and Figure 5). The effect of HbA1c on lowering renal biomarker levels was not significant according to the multiple linear regression analysis (Supplemental File (available here)).

Urine KIM-1 levels decreased in both groups, but the change in the urine KIM-1 level did not differ between groups (−0.28 ± 1.15 ng/mg creatinine in the gemigliptin group vs. −0.5 ± 0.90 ng/mg creatinine in the control group, *P* = 0.156).

## 4. Adverse Events

Adverse events due to gemigliptin were rare. The most commonly reported adverse events in related studies were hypoglycemia, upper respiratory tract infection, urinary tract infection, nasopharyngitis, headache, diarrhea, arthralgia, hypertension, and cough [9]. However, we observed only one adverse event in one patient who reported palpitation after gemigliptin administration. This patient ultimately asked to withdraw from the trial.

## 5. Discussion

Many investigators have studied the pleiotropic properties of DPP-4 inhibitors to highlight their potential benefits in various diseases. The possible mechanisms of DPP-4 inhibition are related to the anti-inflammatory and immunoregulatory effects of DPP-4 inhibitors, reduced cytokine overproduction, enhanced GLP-1 anti-inflammatory activity, and stimulated direct pulmonary anti-inflammatory effects [10–13]. At present, scarce data are available concerning the effects of DPP-4 inhibitors on vascular calcification in vivo. We chose gemigliptin due to its unique characteristics, including its action as a highly competitive and selective DPP-4 inhibitor. Then, we evaluated the effect of gemigliptin on vascular calcification using CAC scores and CAVI. After a follow-up period of six months, the CAC scores and CAVI did not differ between the two groups. CAVI improved in the control and gemigliptin groups but was significantly changed only in the control group. Possibly, 24 weeks of improvement in glucose control may have influenced arterial stiffness in both groups. One other study also indicated that short-term glycemic and blood pressure treatment improved arterial wall stiffness among patients with type 2 diabetes [14]. However, the markers of vascular calcification and bone alkaline phosphatase (BALP) decreased significantly in the gemigliptin group compared with those in the control group. BALP is a sensitive and specific marker for osteoblast activity and bone formation. Alkaline phosphatase stimulates mineralization mainly through the modulation of the balance between inorganic phosphate and inorganic pyrophosphate and plays a role in cardiovascular remodeling. Yan et al. [15] showed that BALP is an independent risk factor for abdominal aortic calcification and suggested a strong relationship between BALP and vascular calcification in a dialysis patient population. Shantouf et al. [16] reported a significant association between serum alkaline phosphatase and coronary artery calcification in maintenance hemodialysis. Taken together, the decrease in BALP and tendency for increased osteopontin levels in response to gemigliptin in our study might indicate a potential role for DPP4 inhibitors regarding long-term protection against vascular calcification.

The role of DPP-4 inhibitors in renal disease is not fully understood. DPP-4 is highly expressed in proximal renal tubular cells and has proteolytic activity via the extracellular catabolism of proteins in the kidney, such as proline-containing peptides. DPP-4 inhibition likely alters the degradation and regulation of peptides in the lumen and thus influences tubular structure and function in diabetes [17]. In addition to the glucose-lowering effects of DPP-4 inhibitors, tissue-protective effects of DPP-4 inhibition have been demonstrated in ischemia-reperfusion injury, DKD, and CKD. Kim et al. [18] reported that gemigliptin treatment led to reduced apoptosis, inflammation, and oxidative stress in a murine model of adriamycin-induced nephropathy. Choi et al. [19] showed that gemigliptin attenuated cisplatin-induced renal dysfunction in mice. The mechanisms were possibly due to inhibition of the apoptotic death of renal tubular cells and inflammatory responses. Of interest, we studied biomarkers of early kidney injury, KIM-1, NGAL, and LFABP, which are not only more sensitive than serum creatinine to identify acute kidney injury but also can indicate specific damage to the proximal tubules [20]. Urine NGAL and urine LFABP levels were significantly decreased after gemigliptin treatment. To the best of our knowledge, this constitutes the first study to show that DPP4 inhibitors alleviate kidney injury by measuring new biomarker levels. Urine NGAL levels have been shown to increase in many pathologic conditions, including DKD [21]. The level of urinary NGAL appears to increase beginning in the early phase of diabetic nephropathy, and the NGAL level is independently associated with albuminuria [22]. The novel finding of this study is that urine NGAL levels significantly decreased after gemigliptin treatment, which may indicate that gemigliptin helps ameliorate tubulointerstitial damage. Indeed, the evolution of the estimated GFR was not significantly changed. A short follow-up time may have influenced the therapeutic response. NGAL is considered a biomarker for monitoring disease progression.

L-FABP regulates fatty acid uptake and intracellular transport, and the excretion rate of this protein is associated with tubulointerstitial structural damage [23]. Therefore, L-FABP levels can be used to identify patients with a high susceptibility to renal stress. Increased urine concentrations of L-FABP have been observed among patients with DKD [24]. Indeed, urine L-FABP levels were elevated at the very early stage of DKD, even before any clinical signs of glomerular damage were detectable, and tubular damage, albuminuria, and end-stage renal disease were independently predicted [25]. In this study, urine L-FABP levels markedly decreased after gemigliptin treatment. These results suggest that gemigliptin can reduce kidney injury and thus reduce the rate of renal disease progression. Long-term prospective follow-up studies may demonstrate whether eGFR is affected.

KIM-1 is a transmembrane protein that is markedly upregulated in the renal proximal tubules after injury [26]. Urinary levels of KIM-1 were significantly elevated among patients with diabetes, indicating the existence of diabetic tubular damage at the early stage of DKD [27]. The expression of KIM-1 is mainly upregulated in proximal tubule cells in both rodents and humans [28]. We found that urine KIM-1 levels decreased significantly in both groups. The difference in the urine KIM-1 level was similar in both groups. However, KIM-1 may not be as sensitive as urine LFABP and NGAL for detecting the favorable effects of gemigliptin. In a related study, gemigliptin helped ameliorate proteinuria along with reducing nephrin [29]. These results were consistent with findings from animal models of diabetic nephropathy in which gemigliptin was shown to protect podocytes. The renoprotective effects of gemigliptin, including reduced albuminuria, may have resulted from inhibiting podocyte injury [6]. The other mechanisms by which gemigliptin significantly reduces renal injury may be related to the antifibrotic, antiapoptotic, and anti-inflammatory action of DPP4 inhibitors independent of their antiproteinuric and glucose-lowering effects. In our study, more patients in the gemigliptin group received ACEi/ARB medications than those in the control group. However, the reduced proteinuria did not significantly differ. We then used other biomarkers that were more sensitive to detect early changes in renal function. The markers that we chose to study were sensitive and established indicators of subtle injury to the kidney before measurable functional decline. These urinary markers were elevated before microalbuminuria was observed; thus, their values might have been altered due to the changes in the estimated GFR.

Our study encountered several limitations. First, patients were followed up with for a relatively short duration (six months). The antiatherosclerotic effects of gemigliptin, such as the effects on the CAC score and CAVI, were not clearly demonstrated. Exposure to the study drug may not have been sufficiently long to reverse the effects of years of proatherosclerotic processes among patients with a median duration of diabetes mellitus of more than 10 years; therefore, we cannot exclude the possibility of either benefit or risk with a longer duration of gemigliptin therapy. A longer study needs to be conducted. Second, the study included a relative difference in baseline urine protein levels and ACEI/ARB prescription rates between the two groups of patients, and only approximately 46 to 60% of the patients received ACEIs/ARBs. This could have interfered with the interpretation of the study results. Finally, the study employed an open-label, randomized controlled design.

In conclusion, our study demonstrated that gemigliptin improved glycemic control, vascular calcification marker levels, and kidney injury biomarker levels. Urinary excretion of these markers is an early, sensitive, and specific marker for DKD that helps demonstrate the beneficial effect of gemigliptin. In addition to the rare side effects associated with this class of DPP-4 inhibitor, their pleiotropic actions help alleviate tubular injury and delay DKD progression, which is of great clinical relevance. Additional large and long-term studies are needed to confirm the clinical benefit and utility of these inhibitors.

## Figures and Tables

**Table 1:** Baseline characteristics of the two groups.

	Gemigliptin (*N* = 94)	Control (*N* = 88)	*P* value
Male (%)	55 (58.5)	52 (59.1)	0.937
Age (years)	62.9 ± 8.7	62.7 ± 10.5	0.881
Duration of diabetes (years)	13 (1-20)	11 (1-40)	0.493
Hypoglycemic agents (%)			0.064
No	3 (3.33)	9 (10.34)	
Yes	87 (96.67)	78 (89.66)	
Hypoglycemic agents (%)			
Insulin (%)	34 (37.78)	37 (42.53)	0.519
Glipizide (%)	64 (71.11)	44 (50.57)	0.005
Pioglitazone (%)	34 (37.78)	29 (33.33)	0.537
Metformin (%)	43 (47.78)	37 (42.53)	0.483
Acarbose (%)	6 (6.67)	1 (1.15)	0.118^†^
Drug method (%)			0.238
Insulin	8 (9.20)	14 (17.95)	
Oral hypoglycemic agent	53 (60.92)	41 (52.56)	
Insulin+oral hypoglycemic agents	26 (29.89)	23 (29.49)	
Hypertensive medications			
ACEi/ARB (%)	57 (63.3)	40 (46.0)	0.020
Calcium channel blockers (%)	55 (61.1)	44 (50.6)	0.158
Beta-blocker (%)	30 (33.3)	38 (43.7)	0.157
Methyldopa (%)	2 (2.2)	5 (5.8)	0.272^†^
Hydralazine (%)	15 (16.7)	27 (31.0)	0.025
Alpha-blocker (%)	12 (13.3)	16 (18.4)	0.357
Body weight (kg)	75.1 ± 16.5	72.7 ± 14.1	0.296
Body mass index (kg/m^2^)	28.5 ± 5.9	27.8 ± 4.7	0.361
Hypertension (%)	78 (83.3)	67 (77.1)	0.495
Dyslipidemia (%)	33 (35.7)	32 (37.1)	0.897
Peripheral vascular disease (%)	0	2 (2.9)	0.455
Ischemic heart disease (%)	6 (7.1)	20 (22.9)	0.500

Data are presented as the mean ± SD or percentage. Chi-square test. ^†^Fisher's exact test. ACEi: angiotensin-converting enzyme inhibitor; ARB: angiotensin receptor blocker.

**Table 2:** Changes in clinical characteristics and laboratory indices in the gemigliptin group from baseline to month 6.

	Baseline	Month 6	Mean difference	*P* value
Body weight (kg)	75.16 ± 16.6	75.37 ± 16.71	0.22 ± 4.20	0.622
Body mass index (kg/m^2^)	28.52 ± 6.01	28.59 ± 5.94	0.07 ± 1.57	0.673
Mean arterial blood pressure	112.08 ± 14.68	108.76 ± 16.59	−3.31 ± 18.02	0.080
Hemoglobin (g/dL)	12.44 ± 1.80	12.17 ± 1.75	−0.26 ± 0.99	0.018
Estimated GFR (mL/min/1.73 m^2^)	46.83 ± 19.14	45.8 ± 21.27	−1.03 ± 10.00	0.340
BUN (mg/dL)	26.32 ± 10.18	27.58 ± 12.99	1.27 ± 8.75	0.189
Serum creatinine (mg/dL)	1.62 ± 0.59	1.70 ± 0.70	0.09 ± 0.33	0.018
Fasting plasma glucose (mg/dL)	185.36 ± 56.91	152.02 ± 60.14	−33.34 ± 59.40	<0.001
Hemoglobin A1C (%)	8.37 ± 1.95	7.7 ± 1.98	−0.67 ± 1.59	<0.001
Serum potassium (mEq/L)	4.49 ± 0.50	4.40 ± 0.48	−0.08 ± 0.49	0.141
Serum magnesium (mg/dL)	1.92 ± 0.30	2.14 ± 1.26	0.22 ± 1.19	0.128
Serum calcium (mg/dL)	9.4 ± 0.52	9.14 ± 0.95	−0.26 ± 1.03	0.037
Serum phosphate (mg/dL)	3.88 ± 0.70	3.75 ± 0.76	−0.14 ± 0.69	0.105
Serum albumin (g/dL)	3.84 ± 0.52	3.87 ± 0.48	0.04 ± 0.35	0.379
Urine protein, 24 hours (g/day)	1.77 ± 3.15	1.76 ± 3.39	−0.01 ± 2.96	0.983
Coronary artery calcium score	655.72 ± 905.88	690.93 ± 932.89	35.21 ± 252.88	0.180
CAVI	9.37 ± 1.35	9.08 ± 1.52	−0.28 ± 1.58	0.085
Serum myeloperoxidase (*μ*g/L)	641.58 ± 232.89	620.76 ± 244.38	−20.82 ± 298.75	0.501
Serum osteopontin (ng/mL)	2438.58 ± 1439.96	2749.25 ± 1506.93	310.67 ± 2222.67	0.179
Serum bone alkaline phosphatase (*μ*g/L)	18.15 ± 11.62	12.30 ± 6.81	−5.85 ± 10.65	<0.001
Urine NGAL (ng/mg creatinine)	387.9 ± 1094.02	316.37 ± 679.96	−71.53 ± 837.30	0.412
Urine Kim (ng/mg creatinine)	0.90 ± 0.97	0.63 ± 0.82	−0.28 ± 1.15	0.023
Urine LFABP (*μ*g/mg creatinine)	91.32 ± 146.92	37.16 ± 68.47	−54.17 ± 141.64	<0.001

Data are presented as the mean ± SD. CAVI: cardio-ankle vascular index; LFABP: liver acid-binding protein; NGAL: neutrophil gelatinase-associated lipocalin; Kim-1: kidney injury molecule-1.

**Table 3:** Changes in clinical characteristics and laboratory indices in the control group from baseline to month 6.

	Baseline	Month 6	Mean difference	*P* value
Body weight (kg)	72.74 ± 14.24	72.51 ± 15.20	−0.23 ± 4.70	0.654
Body mass index (kg/m^2^)	27.72 ± 4.61	27.63 ± 4.92	−0.09 ± 1.75	0.619
Mean arterial blood pressure	115.98 ± 17.41	116.32 ± 19.12	0.34 ± 17.33	0.856
Hemoglobin (g/dL)	12.35 ± 1.94	12.1 ± 1.98	−0.25 ± 0.88	0.012
Estimated GFR (mL/min/1.73 m^2^)	44.27 ± 21.25	42.36 ± 22.36	−1.90 ± 10.91	0.116
BUN (mg/dL)	27.48 ± 13.33	30.28 ± 15.44	2.79 ± 8.30	0.004
Serum creatinine (mg/dL)	1.75 ± 0.69	1.88 ± 0.95	0.14 ± 0.42	0.004
Fasting plasma glucose (mg/dL)	167.19 ± 51.07	154.52 ± 58.65	−12.67 ± 62.46	0.065
Hemoglobin A1C (%)	8.12 ± 1.70	7.98 ± 1.88	−0.15 ± 1.70	0.451
Serum potassium (mEq/L)	4.59 ± 0.75	4.47 ± 0.50	−0.12 ± 0.77	0.183
Serum magnesium (mg/dL)	1.98 ± 0.27	1.92 ± 0.24	−0.05 ± 0.26	0.128
Serum calcium (mg/dL)	9.27 ± 0.51	9.25 ± 0.46	−0.02 ± 0.52	0.729
Serum phosphate (mg/dL)	4.05 ± 0.85	3.95 ± 0.86	−0.10 ± 0.88	0.316
Serum albumin (g/dL)	3.78 ± 0.46	3.74 ± 0.50	−0.04 ± 0.28	0.239
Urine protein, 24 hours (g/day)	0.89 ± 1.60	1.39 ± 2.62	0.49 ± 2.22	0.060
Coronary artery calcium score	729.95 ± 1123.98	744.67 ± 1148.90	14.73 ± 161.66	0.395
CAVI	9.26 ± 1.44	8.69 ± 2.06	−0.57 ± 1.81	0.005
Serum myeloperoxidase (*μ*g/L)	653.06 ± 208.78	655.67 ± 232.65	2.61 ± 295.2	0.934
Serum osteopontin (ng/mL)	2442.31 ± 1368.92	2668.69 ± 1656.47	226.39 ± 2057.35	0.305
Serum bone alkaline phosphatase (*μ*g/L)	15.59 ± 11.06	15.68 ± 8.24	0.08 ± 11.45	0.947
Urine NGAL (ng/mg creatinine)	333.67 ± 627.23	590.95 ± 1252.05	257.28 ± 1047.29	0.024
Urine Kim (ng/mg creatinine)	1.11 ± 1.07	0.61 ± 0.74	−0.50 ± 0.90	<0.001
Urine LFABP (*μ*g/mg creatinine)	48.86 ± 67.14	55.47 ± 86.31	6.6 ± 88.94	0.488

Data are presented as the mean ± SD. CAVI: cardio-ankle vascular index; LFABP: liver acid-binding protein; NGAL: neutrophil gelatinase-associated lipocalin; Kim-1: kidney injury molecule-1.

**Table 4:** Comparison of mean changes in various parameters from baseline to month 6 between the two groups.

Mean change	Gemigliptin (*N* = 94)	Control (*N* = 88)	95% CI	*P* value
Body weight (kg)	0.22 ± 4.20	−0.23 ± 4.70	-0.87, 1.75	0.506
Body mass index (kg/m^2^)	0.07 ± 1.57	−0.09 ± 1.75	-0.33, 0.65	0.512
Mean arterial blood pressure	−3.31 ± 18.02	0.34 ± 17.33	-8.89, 1.59	0.171
Hemoglobin (g/dL)	−0.26 ± 0.99	−0.25 ± 0.88	-0.30, 0.28	0.952
Estimated GFR (mL/min/1.73 m^2^)	−1.03 ± 10.00	−1.90 ± 10.91	-2.29, 4.04	0.587
BUN (mg/dL)	1.27 ± 8.75	2.79 ± 8.30	-4.16, 1.10	0.254
Serum creatinine (mg/dL)	0.09 ± 0.33	0.14 ± 0.42	-0.16, 0.06	0.389
Fasting plasma glucose (mg/dL)	−33.34 ± 59.40	−12.67 ± 62.46	-38.96, -2.38	0.027
Hemoglobin A1C (%)	−0.67 ± 1.59	−0.15 ± 1.70	-1.05, -0.01	0.048
Serum potassium (mEq/L)	−0.08 ± 0.49	0.44 ± 4.02	-0.96, 1.44	0.734
Serum magnesium (mg/dL)	0.22 ± 1.19	−0.05 ± 0.26	-0.01, 0.56	0.057
Serum calcium (mg/dL)	−0.26 ± 1.03	−0.02 ± 0.52	-0.50, 0.02	0.073
Serum phosphate (mg/dL)	−0.14 ± 0.69	−0.10 ± 0.88	-0.29, 0.23	0.800
Serum albumin (g/dL)	0.04 ± 0.35	−0.04 ± 0.28	-0.03, 0.18	0.152
Urine protein, 24 hours (g/day)	−0.01 ± 2.96	0.49 ± 2.22	-1.35, 0.35	0.246
Coronary artery calcium score	35.21 ± 252.88	14.73 ± 161.66	-42.07, 83.03	0.519
CAVI	−0.28 ± 1.58	−0.57 ± 1.81	-0.22, 0.79	0.265
Serum myeloperoxidase (*μ*g/L)	−20.82 ± 298.75	226.39 ± 2057.35	-543.35, 711.91	0.596
Serum osteopontin (ng/mL)	310.67 ± 2,222.67	226.39 ± 2057.35	-543.35, 711.91	0.791
Serum bone alkaline phosphate (*μ*g/L)	−5.84 ± 10.65	0.08 ± 11.45	-9.16, -2.69	<0.001
Urine NGAL (ng/mg creatinine)	−71.53 ± 837.30	257.28 ± 1,047.29	-606.19, -51.43	0.020
Urine Kim (ng/mg creatinine)	−0.28 ± 1.15	−0.50 ± 0.90	-0.08, 0.52	0.156
Urine LFABP (*μ*g/mg creatinine)	−54.17 ± 141.64	6.6 ± 88.94	-95.30, -26.24	0.001

Data are presented as the mean ± SD and 95% CI. CAVI: cardio-ankle vascular index; LFABP: liver acid-binding protein; NGAL: neutrophil gelatinase-associated lipocalin; Kim-1: kidney injury molecule-1.

## Data Availability

The datasets generated and analyzed during the current study are available in the figshare repository (https://figshare.com/s/059283ae1fd803a85939).

## Abbreviations

DKD: Diabetic kidney disease
eGFR: Estimated glomerular filtration rate
KIM-1: Kidney injury molecule-1
L-FABP: Liver-type fatty acid-binding protein
NGAL: Neutrophil gelatinase-associated lipocalin
UACR: Urinary albumin-creatinine ratio
AAC: Abdominal aortic calcification.

## Supplementary Materials

Supplementary Materials Table 1: laboratory data from visit 1 to visit 4 in the gemigliptin group and control group. Table 2: change in urine NGAL after adjustment with HbA1C in the gemigliptin group. Table 3: change in urine Kim-1 after adjustment with HbA1C in the gemigliptin group. Table 4: change in urine LFABP after adjustment with HbA1C in the gemigliptin group. Table 5: change in serum bone alkaline after adjustment with HbA1C in the gemigliptin group. Table 6: change in eGFR after adjustment with HbA1C in the gemigliptin group.
