# Supplementary material for: Effect of Dipeptidyl Peptidase-4 (DPP-4) Inhibition on Biomarkers of Kidney Injury and Vascular Calcification in Diabetic Kidney Disease: A Randomized Controlled Trial
Source: J Diabetes Res. 2021 Oct 16;2021:7382620. doi: 10.1155/2021/7382620 (PMC8541867; doi:10.1155/2021/7382620)
Supplement: Supplementary Materials — Table 1: laboratory data from visit 1 to visit 4 in the gemigliptin group and control group. Table 2: change in urine NGAL after adjustment with HbA1C in the gemigliptin group. Table 3: change in urine Kim-1 after adjustment with HbA1C in the gemigliptin group. Table 4: change in urine LFABP after adjustment with HbA1C in the gemigliptin group. Table 5: change in serum bone alkaline after adjustment with HbA1C in the gemigliptin group. Table 6: change in eGFR after adjustment with HbA1C in the gemigliptin group. [file 7382620.f1.docx]

Supplemental File

**Table 1s:**Lab visit 1 to visit 4 (BUN | Cr | FBS | eGFR)

|  | | |  |  |  |  |  |
| --- | --- | --- | --- | --- | --- | --- | --- |
|  | n | Visit 1 | Visit 2 | Visit 3 | Visit 4 | p-value* | p-value** |
|  |  | Mean ± SD | Mean ± SD | Mean ± SD | Mean ± SD |  |  |
| **BUN** |  |  |  |  |  |  |  |
| Group A | 70 | 26.49 ± 10.00 | 27.41 ± 11.88 | 26.96 ± 12.66 | 27.74 ± 13.24 | 0.564 | 0.138 |
| Group B | 68 | 28.99 ± 13.45 | 31.19 ± 18.38 | 29.11 ± 14.09 | 32.09 ± 15.71 | 0.058 |  |
| **Cr** |  |  |  |  |  |  |  |
| Group A | 79 | 1.60 ± 0.61 | 1.63 ± 0.64 | 1.66 ± 0.65 | 1.69 ± 0.73 | 0.048 | 0.047 |
| Group B | 74 | 1.80 ± 0.71 | 1.89 ± 0.83 | 1.89 ± 0.89 | 1.95 ± 0.98 | 0.010 |  |
| **FBS** |  |  |  |  |  |  |  |
| Group A | 80 | 188.54 ± 57.30 | 149.47 ± 48.51 | 154.59 ± 69.16 | 152.54 ± 61.83 | <0.001 | 0.657 |
| Group B | 75 | 171.11 ± 52.23 | 167.93 ± 66.31 | 163.08 ± 60.16 | 155.15 ± 54.93 | 0.249 |  |
| **eGFR** |  |  |  |  |  |  |  |
| Group A | 74 | 47.35 ± 19.93 | 47.17 ± 21.14 | 46.45 ± 20.98 | 46.18 ± 21.65 | 0.505 | 0.071 |
| Group B | 70 | 41.37 ± 20.10 | 40.13 ± 20.28 | 40.71 ± 20.64 | 40.39 ± 21.69 | 0.558 |  |

*One- way repeated measure ANOVA
**Two- way repeated measureANOVA

Significantif p<0.05

**Table 2s:**Change in urine NGAL after adjustment with HbA1C in Gemigliptin group

Adjusted △ HbA1C with △Urine NGAL ratio

|  | Univariable analysis | | | Multivariable analysis | | |
| --- | --- | --- | --- | --- | --- | --- |
|  | B | 95%CI | p-value | B | 95%CI | p-value |
| Gemigliptin | -328.813 | -606.194 , -51.431 | 0.020 | -347.296 | -677.294 , -17.297 | 0.039 |
| △ HbA1C | 57.938 | -41.489 , 157.365 | 0.251 | 41.596 | -57.985 , 141.177 | 0.410 |

Multiple linear regression

Significant if p<0.05

**Table 3s:** Change in urine Kim1 after adjustment with HbA1C in Gemigliptin group

Adjusted △ HbA1C with △ Urine Kim1

|  | | | | | |  | |  |  |  | |
| --- | --- | --- | --- | --- | --- | --- | --- | --- | --- | --- | --- |
|  | | Univariable analysis | | | | | | Multivariable analysis | | | |
|  | | B | 95%CI | | | p-value | | B | 95%CI | p-value | |
| Gemigliptin | | 0.22 | -0.085 , 0.525 | | | 0.156 | | 0.288 | -0.017 , 0.593 | 0.064 | |
| △ HbA1C | | -0.032 | -0.124 , 0.059 | | | 0.487 | | -0.019 | -0.111 , 0.073 | 0.685 | |
|  |  | | |  |  | |  |  |  |  |  |
| Multiple linear regression  Significant if p<0.05 | | | | | | | | | | |  |
|  | | | | | | | | | | |  |

**Table 4s:** Change in urine LFABP after adjustment with HbA1C in Gemigliptin group

Adjusted △ HbA1C with △ Urine LFABP

|  | | |  |  |  |  |
| --- | --- | --- | --- | --- | --- | --- |
|  | Univariable analysis | | | Multivariable analysis | | |
|  | B | 95%CI | p-value | B | 95%CI | p-value |
| Gemigliptin | -60.772 | -95.688 , -25.855 | 0.001 | -48.067 | -81.979 , -14.156 | 0.006 |
| △ HbA1C | 4.987 | -5.345 , 15.32 | 0.342 | 2.726 | -7.508 , 12.959 | 0.599 |

Multiple linearregression

|  |
| --- |

**Table 5s:** Change in serum bone alkaline after adjustment with HbA1C in Gemigliptin group

Adjusted △ HbA1C with △ Bone alkaline phosphate (pg/mL)

|  | |  | |  |  |  |  |  |  |
| --- | --- | --- | --- | --- | --- | --- | --- | --- | --- |
|  | Univariable analysis | | | | | | Multivariable analysis | | |
|  | B | | 95%CI | | | p-value | B | 95%CI | p-value |
| Gemigliptin | -5927.192 | | -9158.853 , -2695.532 | | | <0.001 | -6035.068 | -9592.794 , -2477.341 | 0.001 |
| △ HbA1C | -1046.109 | | -2143.008 , 50.789 | | | 0.061 | -1336.349 | -2412.062 , -2606.36 | 0.015 |

| Multiple linear regression  Significant if p<0.05 |
| --- |

**Table 6s:** Change in eGFR after adjustment with HbA1C in Gemigliptin group

|  | Univariable analysis | | | Multivariable analysis | | |
| --- | --- | --- | --- | --- | --- | --- |
|  | B | 95%CI | p-value | B | 95%CI | p-value |
| Gemigliptin | 0.873 | -2.294 , 4.039 | 0.587 | 0.833 | -2.213 , 3.878 | 0.590 |
| △ HbA1C | 0.444 | -0.455 , 1.343 | 0.331 | 0.490 | -0.427 , 1.407 | 0.293 |
| Multiple linear regression | | |  |  |  |  |
| Significant if p<0.05 | |  |  |  |  |  |

dx.doi.org/10.17504/protocols.io.brvtm66n
